# Preoperative prediction of tumor budding in rectal cancer using multiple machine learning algorithms based on MRI T2WI radiomics

**DOI:** 10.3389/fonc.2023.1267838

**Published:** 2023-10-24

**Authors:** Xueting Qu, Liang Zhang, Weina Ji, Jizheng Lin, Guohua Wang

**Affiliations:** ^1^ Department of Radiology, Qingdao Municipal Hospital, Qingdao University, Qingdao, Shandong, China; ^2^ Department of Radiology, the Affiliated Hospital of Qingdao University, Qingdao, Shandong, China

**Keywords:** tumor budding 3, rectal cancer, magnetic resonace imaging, algorithm, radiomics

## Abstract

**Objective:**

This study aimed to explore the radiomics model based on magnetic resonance imaging (MRI) T2WI and compare the value of different machine algorithms in preoperatively predicting tumor budding (TB) grading in rectal cancer.

**Methods:**

A retrospective study was conducted on 266 patients with preoperative rectal MRI examinations, who underwent complete surgical resection and confirmed pathological diagnosis of rectal cancer. Among them, patients from Qingdao West Coast Hospital were assigned as the training group (n=172), while patients from other hospitals were assigned as the external validation group (n=94). Regions of interest (ROIs) were delineated, and image features were extracted and dimensionally reduced using the Least Absolute Shrinkage and Selection Operator (LASSO). Eight machine algorithms were used to construct the models, and the diagnostic performance of the models was evaluated and compared using receiver operating characteristic (ROC) curves and the area under the curve (AUC), as well as clinical utility assessment using decision curve analysis (DCA).

**Results:**

A total of 1197 features were extracted, and after feature selection and dimension reduction, 11 image features related to TB grading were obtained. Among the eight algorithm models, the support vector machine (SVM) algorithm achieved the best diagnostic performance, with accuracy, sensitivity, and specificity of 0.826, 0.949, and 0.723 in the training group, and 0.713, 0.579, and 0.804 in the validation group, respectively. DCA demonstrated the clinical utility of this radiomics model.

**Conclusion:**

The radiomics model based on MR T2WI can provide an effective and noninvasive method for preoperative TB grading assessment in patients with rectal cancer.

## 1 Introduction

Colorectal cancer (CRC) ranks second in terms of cancer-related deaths in the United States and is a leading cause of death for men under the age of 50 (1). The incidence of rectal cancer accounts for approximately a third of all cases (2). The treatment and prognosis assessment of rectal cancer primarily rely on TNM staging. However, patients with the same pathological stage may exhibit significantly different clinical treatment outcomes (3). Exploring additional factors that influence tumor prognosis can provide better guidance and treatment plans for patients, optimize patient management, and improve survival quality (4–6). It has been shown that perineural invasion, tumor deposits, and tumor budding are all adverse prognostic factors (5, 7, 8).

TB,as a process of epithelial-mesenchymal transition in tumors (9, 10), was recommended by the International Tumor Budding Consensus Conference (ITBCC) in 2016 to be included in the guidelines/protocols and staging systems for rectal cancer pathology reporting. It also provided a definition for tumor budding (11). TB refers to the presence of scattered tumor cells or small clusters of tumor cells with poor differentiation at the invasive front of the tumor, observed under high-power microscopy. These cell groups are often single tumor cells or comprise less than 5 cells. TB is categorized into three grades based on the number of buds: low-grade budding (Budding1, Bd1) with 0-4 buds, intermediate-grade budding (Bd2) with 5-9 buds, and high-grade budding (Bd3) with ≥10 buds (within a 20x visual field, corresponding to an area of 0.785 mm²). High-grade budding has been significantly associated with reduced overall survival rate, adverse clinical-pathological features, invasive growth patterns, lymph node metastasis and tumor recurrence rate (4, 8) (11–14). The AJCC/UICC cancer staging guidelines have included tumor budding as an additional prognostic factor for rectal cancer (9). However, as a postoperative pathological characteristic, it cannot provide optimal assistance for preoperative surgical planning. Therefore, exploring preoperative tumor budding assessment methods is beneficial for implementing more accurate personalized treatment and improving tumor prognosis in a clinical setting.

The diagnosis of rectal cancer plays a crucial role in medical imaging, among which MRI has been recommended as the preferred method for diagnosing rectal cancer due to its excellent soft tissue resolution (3, 6, 15). However, traditional imaging diagnostics still have their limitations. With the advancement of scientific technology, artificial intelligence (AI) has also entered the field of medicine. Multiple studies have shown that AI-based imaging learning models can better predict tumor treatment response and prognosis without relying on pathological reports, thereby supporting accurate determination of clinical treatment plans (16–18). Radiomics is one of the AI imaging learning models (19) that has been used for preoperative TN staging prediction, gene typing, assessment of high-risk tissue histopathological variables, treatment efficacy, and prognosis evaluation in tumors (20–22). Current research on radiomics in rectal cancer mainly focuses on predicting aspects such as complete remission after neoadjuvant therapy for advanced rectal cancer, lymph node metastasis, KRAS/NRAS gene mutations, and microsatellite instability (MSI). Few studies have reported on predicting tumor budding. In recent years, some scholars have begun to explore this area (23, 24). Currently, only Li et al. have conducted a study using T2WI, DWI, and contrast-enhanced imaging to predict tumor budding degree, with respective values for AUC, sensitivity, specificity, and accuracy of 0.796, 92.7%, 65.8%, and 81.2% (25). However, some patients are unable to undergo contrast-enhanced examinations due to issues such as contrast agent allergies, renal function abnormalities, or economic constraints. Therefore, this study aims to utilize various machine learning models based on T2WI radiomics to predict the degree of tumor budding in rectal cancer preoperatively. It also seeks to explore clinical risk factors in order to provide assistance in selecting preoperative surgical approaches for rectal cancer and improving patient prognosis.

## 2 Materials and methods

### 2.1 Participants

**Figure 1:**
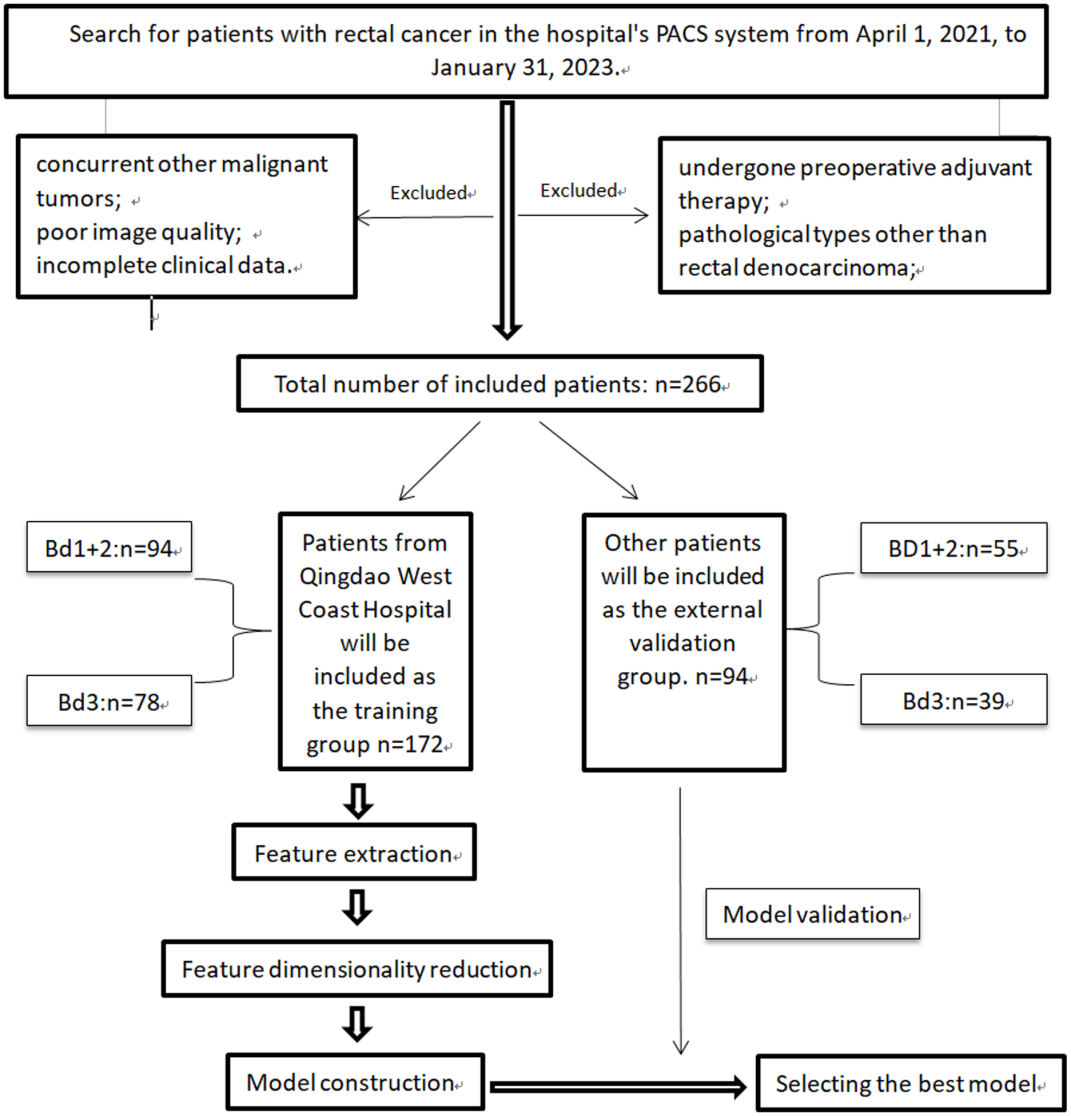
The study flow and overview.

Patients who underwent rectal cancer surgery and parallel MRI scans from April 2021 to April 2023 were included in this study. The study flow and overview are shown in **Figure 1**. Patients from Qingdao West Coast Hospital were assigned as the training group, while patients from Qingdao Municipal Hospital and Qingdao University Affiliated Hospital were assigned as the validation group. The inclusion criteria were as follows (1): Pathological diagnosis of rectal adenocarcinoma with tumor budding grading (2); All patients underwent surgical treatment (3); Preoperative MRI scans performed within 2 weeks (4); Distance of the tumor’s lower margin from the anal verge less than 15cm (26) (5); Complete clinical data. The exclusion criteria were as follows (1): Patients who received preoperative adjuvant therapies such as radiotherapy or immunotherapy (2); Exclusion of patients with mucinous adenocarcinoma6 (3); Patients with concurrent other malignant tumors (4); Patients with poor image quality (5); Patients with incomplete clinical data.

### 2.2 Clinical data

Clinical data collection primarily includes age, gender, carcinoembryonic antigen (CEA), carbohydrate antigen-199 (CA 199), distance from the tumor to the anal verge, length, depth of infiltration, circumferential growth, MR TN staging, distant metastasis, circumferential resection margin (CRM), and extramural vascular invasion (EMVI).

### 2.3 Image acquisition

All patients underwent imaging using 3.0T MR scanners after fasting for at least 4 hours. Conventional axial T2WI images were used to establish the radiomics model in this study. Detailed scanning parameters are provided in **Supplementary Table 1**.

### 2.4 Pathological evaluation

Avoid bias in tumor budding assessment, samples obtained by complete resection of the tumor lesion during surgery were used. The excised samples were stained with Hematoxylin and Eosin (H&E). According to the ITBCC (2016) recommendations and the AJCC/UICC Cancer Staging Guidelines (8th edition, 2017), the pathology report needs to include TN staging, tumor budding grading, pathological type, degree of differentiation, perineural invasion, vascular emboli, circumferential resection margin (CRM). This is necessary for data comparison and analysis in the study. In this research, tumor budding degree was categorized into the intermediate-low-grade group (Bd 1&2) and the high-grade group (Bd 3).

### 2.5 Image segmentation

The DICOM raw images were imported into the open-source software 3D Slicer (https://www.slicer.org/, version5.0.3). One attending physician with 8 years of experience in abdominal MRI diagnosis, serving as the annotating physician, performed lesion segmentation on the axial T2WI images in conjunction with the T2WI sagittal and DWI sequences. The regions of interest were manually outlined along the tumor edges layer by layer, and the software platform automatically reconstructed the volumes of interest (VOI). During the outlining process, one attending physician performs image segmentation along the outermost edge of the tumor visible in the T2WI images, while another senior physician reviews the ROI. When the tumor involves adjacent solid organs, draw along the maximum edge of the tumor to exclude the normal structures of other organs as much as possible. It is difficult to determine whether the nodule around the tumor is a tumor nodule or lymph node metastasis through imaging. When the nodule is closely connected to the tumor and the angle formed by the edges of the nodule and the tumor is obtuse, we believe that the nodule is a part of the tumor. On the contrary, if it is an acute angle, the nodule should be excluded. Any disagreements were resolved through consultation and discussion by two senior physicians. The following considerations were taken into account: (a) Exclusion of the intestinal lumen and its contents; (b) Exclusion of adjacent mesenteric fat tissue; (c) Exclusion of the uninvolved rectal wall. Furthermore, to evaluate the stability of all image features, 30 randomly selected cases were independently segmented by the two physicians again after a one-month interval. Inter- and intraobserver consistency evaluations were conducted (25). The image segmentation process was performed in a single-blind manner, with the two physicians unaware of the tumor budding grading information of the patients.

### 2.6 Radiomics feature extraction and model construction

#### 2.6.1 Image preprocessing

The segmented images obtained from the image segmentation process were imported into the Pyradiomics package (https://github.com/Radiomics/pyradiomics). Due to the presence of various scanning devices and imaging parameters, image preprocessing was required. The following steps were followed for image preprocessing (21, 27):Step 1: N4 bias field correction, which aims to eliminate magnetic field bias or intensity inhomogeneity in the images. Step 2: Resampling of the images using B-spline interpolation to standardize the voxel size to 1mm^3^. Step 3: As per the Image Biomarker Standardization Initiative (IBSI) guidelines (28), grayscale normalization was performed by scaling the gray values of MRI images based on µ ± 3σ (µ: mean gray level within the VOI; σ: gray standard deviation) to minimize MRI signal intensity variations. This normalization step helps eliminate the influence of different field strengths and device parameters on the images.Image preprocessing reduces the effects caused by variations in image acquisition and parameters, ensuring the generalizability and robustness of the constructed models.

#### 2.6.2 Feature extraction

After image preprocessing, image feature extraction for the training group is performed on the aforementioned platform. Features can be divided into three groups: (I) first-order statistics, (II) shape attributes, and (III) texture features. First-order statistics are features extracted directly from the raw data or image, typically including basic statistical measures to provide a basic description of the data. Shape attributes describe the external shape and structure of an object or region, relating to its contour, boundaries, and geometric properties. They are useful in distinguishing different objects, detecting edges and contours, and performing object recognition tasks. Texture features describe the second-order and higher-order spatial distribution of patterns or intensities. They provide information about the texture in the image, such as features related to repetition, variation, and local structure. In this case, several different methods are used for texture feature extraction, including Gray-Level Co-occurrence Matrix (GLCM), Gray-Level Run Length Matrix (GLRLM), Gray-Level Size Zone Matrix (GLSZM), Neighboring Gray Tone Difference Matrix (NGTDM), and Gray-Level Dependence Matrix (GLDM).

Normalization using the z-score method was performed to ensure that all features have a mean of 0 and a standard deviation (SD) of 1, as described in previous studies (24). Feature selection, dimensionality reduction, and prediction model construction were conducted using the feature data from the training group. The feature data from the external validation group were used to validate the effectiveness and performance of the constructed models.

#### 2.6.3 Feature selection and dimensionality reduction

To ensure the robustness of the selected radiomics features, feature dimensionality reduction is performed. Firstly, the interclass/intraclass correlation coefficients (ICCs) are calculated. Features with ICC values greater than 0.75 are selected to exclude those with significant inter-observer/intra-observer variability. Statistical tests such as t-tests or Mann-Whitney U tests are then applied to retain features with p-values less than 0.05. To determine the correlation between features that exhibit high repetitiveness, the Spearman’s rank correlation coefficient can be used. In this method, we calculate the correlation coefficient between features and retain one feature from any pair with a correlation coefficient greater than 0.9 (29, 30). To filter features and retain their descriptive capability to the maximum extent, we can use a greedy recursive elimination strategy. We can employ a nonlinear regression algorithm called LASSO regression, which utilizes the minimum absolute shrinkage and selection operator. By adjusting the weight parameter λ, we can shrink all regression coefficients towards zero. Through 10-fold cross-validation, we can find the optimal value for λ, and retain the features with non-zero coefficients, combining them into a radiomics signature. Finally, we can utilize the scikit-learn package in Python to build the LASSO regression model and calculate the radiomics score (Rad-score) for each patient by linearly combining the retained features. Prediction model building.

**Figure 2:**
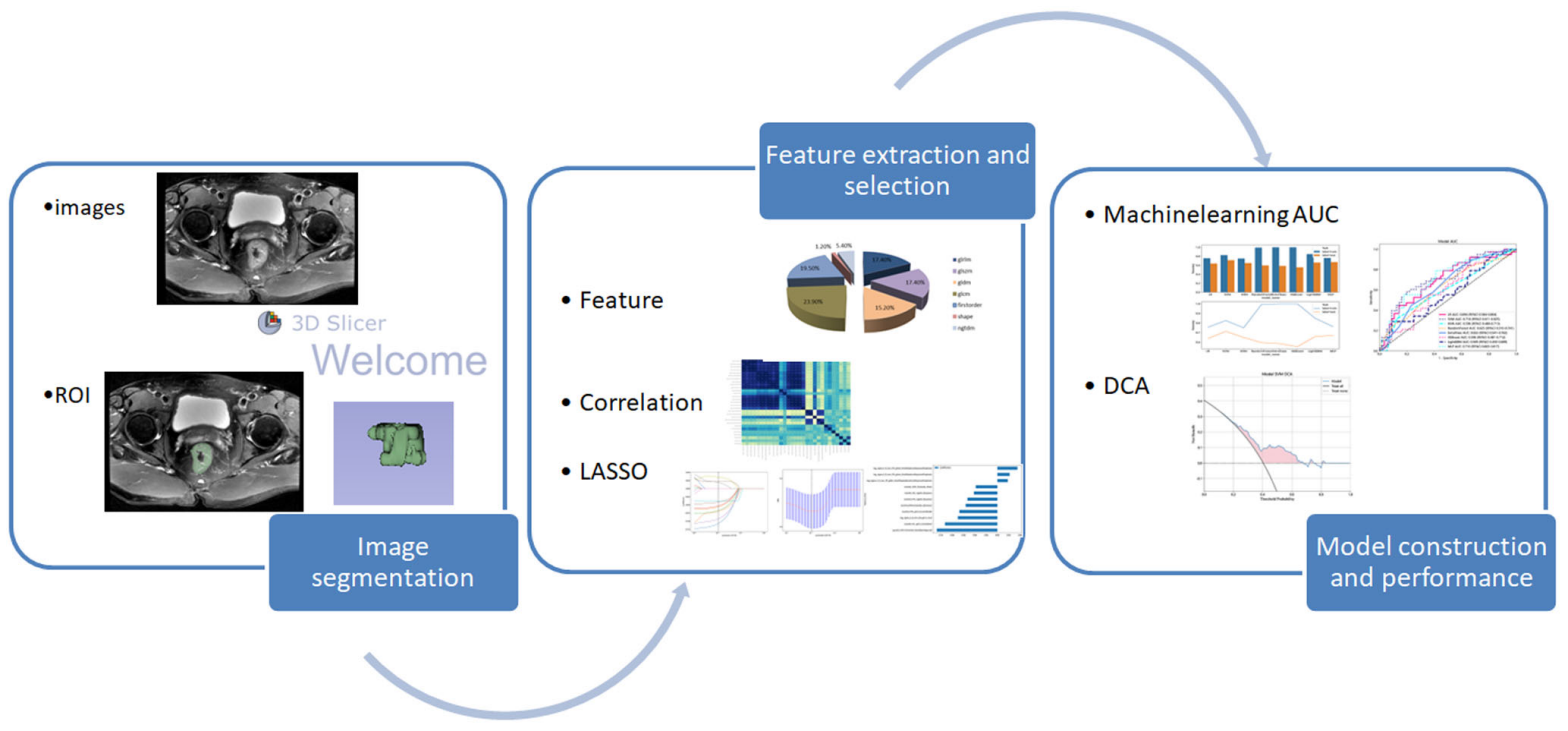
Workflow for building the radiomics model.

The selected radiomic features are individually used with eight classification algorithms: Logistic Regression (LR), K-Nearest Neighbor (KNN), Extra Trees (ET), Random Forest (RF), eXtreme Gradient Boosting (XGBoost), Support Vector Machines (SVM), Light Gradient Boosting Machine (LightGBM), and Multilayer Perceptron (MLP). These eight algorithms are used to build models. The best machine learning algorithm model is selected by calculating the area under the ROC curve. 5-fold cross-validation is employed to obtain the final Rad signature. Please refer to **Figure 2** for the radiomics workflow.

### 2.7 Clinical risk factors

Statistical analysis was performed on clinical variables(age, gender, CEA, CA199 levels), tumor distance from the anal verge, length, depth of invasion, circumferential growth, MRI TN stage, distant metastasis, circumferential margin, and extramural vascular invasion. Independent sample t-tests, Mann-Whitney U-tests, or χ2 tests were used to analyze these variables. Clinically significant risk factors that exhibited a statistically significant difference (P < 0.05) in predicting tumor budding grade were identified.

### 2.8 Statistical analysis

Statistical analysis was performed using Python 3.8 (https://www.python.org) and SPSS 19.0 software. Continuous data in patient clinical information were tested for normality and homogeneity of variance. For comparisons, either t-tests or rank-sum tests were used. Categorical data were compared using the chi-square test.In the training group, radiomic features were first transformed to a normal distribution using regularization. Then, t-tests (when homogeneity of variance) and Mann-Whitney U-tests (when heterogeneity of variance) were conducted to compare the features and identify statistically significant differences.Single-factor and multi-factor logistic regression analyses were performed to evaluate clinical risk factors associated with high-grade tumor budding. Factors with a significance level of P < 0.05 were considered statistically significant. ROC and AUC were used to evaluate the predictive performance of each model in the training group and the external validation group.DCA was used to evaluate the clinical application value of the model.

## 3 Results

### 3.1 Characteristics of the patients

#### 3.1.1 Clinical features

A total of 266 cases were included in the study, with 172 cases in the training group and 94 cases in the external validation group. The clinical characteristics of all patients in the training and external validation groups are shown in **Supplementary Table 2**. Among the training group, 78 cases (45.35%) were classified as high-grade TB, while 38 cases (40.43%) in the external validation group were classified as high-grade TB. No clinical risk factors related to high-grade TB were found (P>0.05).

#### 3.1.2 Radiomics features

**Figure 3:**
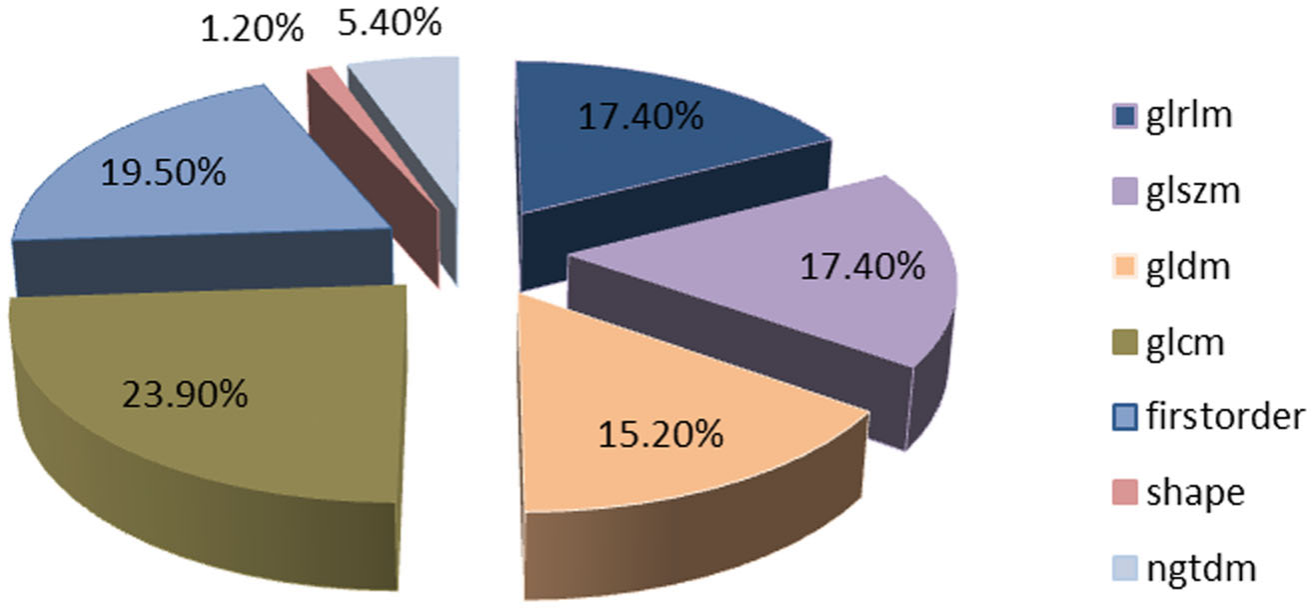
Number and ratio of handcrafted features.

A total of 1197 features were extracted, detailed information can be found in **Table 1**; **Figure 3**. They are mainly divided into three categories (1): First-order statistics (n=234 cases) (2); Shape attributes (n=14 cases) (3); Texture features (n=949 cases), including GLCM, GLRLM, GLSZM, GLDM, and NGTDM.

**Table 1:** Feature extraction and classification.

Feature Type	Subtype	Number of Features
Firstorder	–	234
Texture	GLCM	286
	GLDM	182
	GLRLM	208
	GLSZM	208
	NGTDM	65
Shape	–	14
Total	–	1197

–, no value.

**Figure 4:**
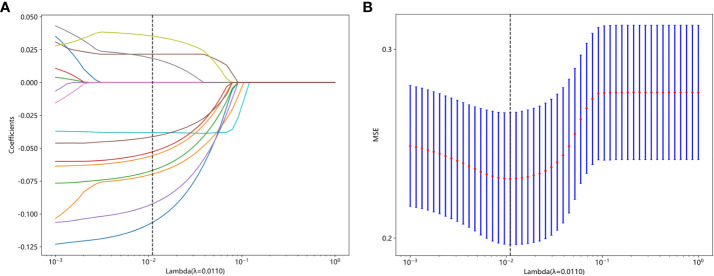
**(A)** The coefficient analysis of LASSO feature selection under 10-fold cross-validation, **(B)** MSE of 10 fold cross validation.

**Figure 5:**
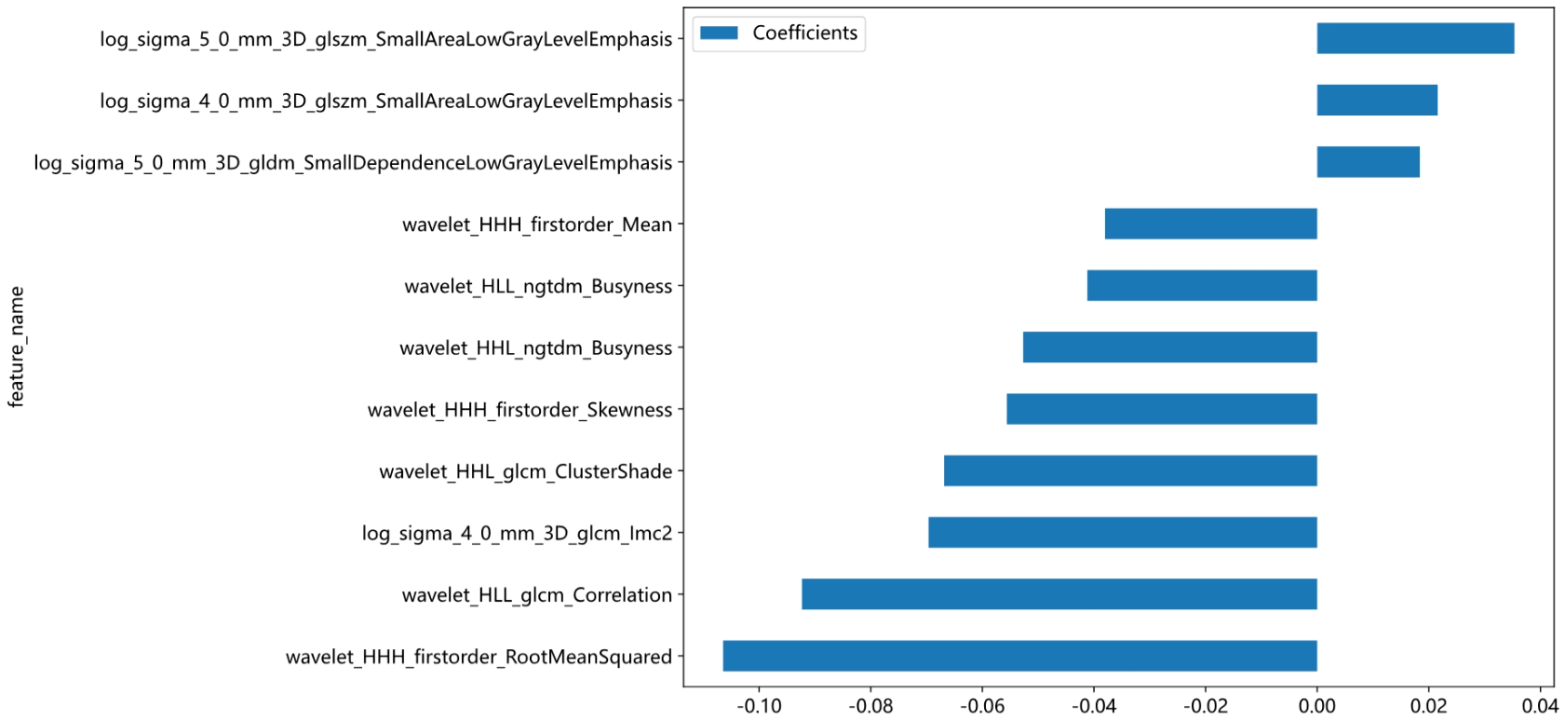
The histogram of the Rad-score based on the selected features.

Features were selected based on group-level and intra-group ICC>0.75. The t-test or Mann-Whitney U test was applied for screening, selecting features with P<0.05, resulting in 29 features being retained. The Spearman rank correlation coefficient was then used to keep only one feature from each pair of features with a correlation coefficient greater than 0.9. Next, a greedy recursive elimination strategy was employed to remove the feature with the highest redundancy at each step, resulting in only 23 features being retained. Pearson correlation coefficient was used to further screen and keep 16 features. LASSO was applied to select features with coefficients greater than 0, resulting in a final retention of 11 features, as shown in **Figure 4**. The detailed feature names and their corresponding Rad-score values can be found in **Figure 5**. The calculation formula of Rad-score is as follows:


$$\begin{array}{l} \text{Rad}-\text{score}=0.45348837209300424-0.069649*\log\_\text{sigma}\_4\_0\_\text{mm}\_3\text{D}\_\text{glcm}\_\text{Imc}2\\ +0.021591*\log\_\text{sigma}\_4\_0\_\text{mm}\_3\text{D}\_\text{glszm}\_\text{SmallAreaLowGrayLevelEmphasis}\\ \begin{array}{l} +0.018396*\log\_\text{sigma}\_5\_0\_\text{mm}\_3\text{D}\_\text{gldm}\_\text{SmallDependenceLowGrayLevelEmphasis}\\ +0.035346*\log\_\text{sigma}\_5\_0\_\text{mm}\_3\text{D}\_\text{glszm}\_\text{SmallAreaLowGrayLevelEmphasis}\\ -0.038032*\text{wavelet}\_\text{HHH}\_\text{firstorder}\_\text{Mean}\\ -0.106451*\text{wavelet}\_\text{HHH}\_\text{firstorder}\_\text{RootMeanSquared} \end{array}\\ -0.055620*\text{wavelet}\_\text{HHH}\_\text{firstorder}\_\text{Skewness} \end{array}$$

### 3.2 Construction and diagnostic performance comparison of radiomics models

**Figure 6:**
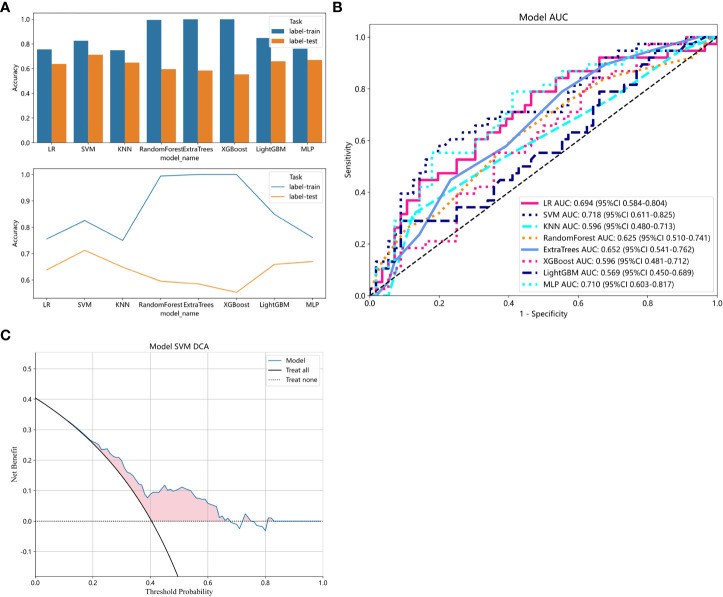
**(A)** Comparison of accuracy among all models. **(B)** ROC analysis of different models on the validation set. **(C)** The decision curve of the SVM model. The y-axis represents standardized net benefit. The blue curve represents the radiomics model. The black curve represents the hypothetical scenario where all patients are successfully predicted. The black dashed line represents the hypothetical scenario where no patients are successfully predicted.

Radiomics models were constructed using eight different classification algorithms. The AUC values for the training group ranged from 0.807 to 1.000, while for the external validation group, they ranged from 0.569 to 0.718. Among all the algorithms, the SVM algorithm demonstrated the best diagnostic performance, with AUC values of 0.892 and 0.718 for the training and external validation groups, respectively (95% CI: 0.8413-0.9420, 0.6111-0.8246). Furthermore, the SVM model showed excellent performance in both the training and external validation groups, achieving better diagnostic accuracy with sensitivity, specificity, and accuracy of 57.9%, 80.4%, and 71.3%, respectively. Detailed data can be found in **Table 2**; **Figure 6** illustrates the accuracy of all models and the AUC values of each rad signature model on the test set. The DCA demonstrated that the SVM model yielded the high net benefit for predicting rectal cancer TB.

**Table 2:** Performance of machine learning classifiers for predicting TB status.

Model name	Task	Accuracy	AUC	95% CI	Sensitivity	Specificity	PPV	NPV
LR	train	0.756	0.807	0.7423 - 0.8720	0.756	0.755	0.720	0.789
LR	test	0.638	0.694	0.5840 - 0.8041	0.789	0.545	0.536	0.789
SVM	train	0.826	0.892	0.8413 - 0.9420	0.949	0.723	0.740	0.944
SVM	test	0.713	0.718	0.6111 - 0.8246	0.579	0.804	0.667	0.738
KNN	train	0.750	0.841	0.7863 - 0.8962	0.731	0.766	0.722	0.774
KNN	test	0.649	0.596	0.4796 - 0.7126	0.316	0.925	0.632	0.653
RF	train	0.994	0.999	0.9969 - 1.0000	1.000	0.989	0.987	1.000
RF	test	0.596	0.625	0.5102 - 0.7408	0.605	0.600	0.500	0.687
ET	train	1.000	1.000	1.0000 - 1.0000	1.000	1.000	1.000	1.000
ET	test	0.585	0.652	0.5413 - 0.7618	0.789	0.455	0.492	0.758
XGBoost	train	1.000	1.000	1.0000 - 1.0000	1.000	1.000	1.000	1.000
XGBoost	test	0.553	0.596	0.4810 - 0.7116	0.842	0.364	0.471	0.769
LightGBM	train	0.849	0.908	0.8653 - 0.9511	0.859	0.840	0.817	0.878
LightGBM	test	0.660	0.569	0.4496 - 0.6886	0.289	0.911	0.687	0.654
MLP	train	0.762	0.828	0.7674 - 0.8889	0.872	0.670	0.687	0.863
MLP	test	0.670	0.710	0.6029 - 0.8173	0.789	0.600	0.566	0.805

AUC, area under curve, CI, confidence interval, PPV, positive predictive value, NPV, negative predictive value, RF, RandomForest, ET, ExtraTrees.

## 4 Discussion

Due to the heterogeneity of tumors, the clinical treatment outcomes and prognosis of rectal cancer can vary significantly. Multiple studies have explored various prognostic factors for tumors, such as histological type, infiltration pattern, differentiation degree, MSI, Aquaporins (AQP-1) (4, 5) thrombus formation, peripheral nerve invasion, tumor deposits, tumor budding, etc. However, most of these factors are based on invasive pathological reports and cannot be identified macroscopically. It poses a great challenge to non-invasive examination methods to determine the pathological prognostic factors of tumors before surgery. The emergence of new technologies like radiomics have provided possibilities in this regard. Many scholars have conducted value assessments of preoperative diagnosis of pathological prognostic factors using radiomics. For example, Li et al. found that a multi-modal MRI radiomics model can predict MSI in rectal cancer preoperatively (AUC values of 0.78 in both the training and validation sets) (31). Chen et al. found that a radiomics model and its nomogram can improve the predictive efficiency of AQP-1 overexpression (AUC values above 0.80 in both the training and validation sets) (32).

As a novel tumor prognostic factor, TB is gradually being recognized for its importance in personalized cancer treatment management. High levels of TB are considered significantly associated with adverse outcomes in patients (10, 33). The AJCC/UICC Cancer Staging Guidelines have included TB as an additional prognostic factor for rectal cancer (8, 9).

Currently, there are few reported studies on the preoperative non-invasive assessment of TB grading. Chen et al. utilized b-value threshold maps and ADC maps to evaluate the preoperative predictive value of TB grading and found that b-value threshold maps performed better than ADC values in assessing TB grading (34). However, b-value threshold maps are not part of the routine MRI imaging sequence, and their image resolution is inferior to T2WI sequences. Due to their status as special sequences, their application is limited. Li et al. developed a radiomics model based on MR plain and enhanced scans to predict TB grading, with an AUC value of 0.79625, demonstrating the feasibility of radiomics in preoperative TB grading prediction. However, in clinical practice, the model’s applicability is somewhat limited by the inability of some patients to undergo the complete sequence acquisition due to issues such as examination costs, contrast agent allergies, and impaired renal function.

The T2WI sequence, as a routine sequence in MRI examinations, not only provides clearer visualization of lesion details compared to b-value threshold maps but also has lower scanning costs compared to dynamic contrast-enhanced sequences. It does not require consideration of factors such as impaired renal function or contrast agent allergies, making it more clinically applicable. Moreover, radiomics models can identify pathological features that are indistinguishable to the naked eye through complex machine algorithms. Therefore, this study is based on the T2WI sequence and utilizes various machine learning methods to explore the optimal radiomics model for predicting tumor budding. This research aims to provide effective assistance in developing appropriate treatment plans and improving patient prognosis.

In this study, we established multiple radiomics predictive models based on T2WI and found that the SVM model exhibited a higher AUC value in predicting TB in rectal cancer. This indicates that the predictive model has good accuracy and stability. As a non-invasive examination method, it can provide assistance in preoperative treatment planning and prognosis assessment, contributing to personalized medical management for rectal cancer patients. The accuracy, sensitivity, and specificity of this model in the training group were 0.826, 0.949, and 0.723, respectively. In the validation group, these values were 0.713, 0.579, and 0.804, respectively. The model showed relatively lower sensitivity but higher specificity and accuracy, which may be attributed to the limited number of high-grade TB samples in the external validation group. In our research, we aimed to enhance the model’s credibility by selecting an external validation group while choosing radiomics verification methods. We then screened out 11 key features and constructed a radiomics model for predicting TB grading. Among these features, texture features were the most abundant (8/11), which is related to the arrangement and distribution patterns of medical image pixels. In MR images, the differences in tissue composition and internal tumor structures provide more detailed variations in local texture features.This is also one of the reasons for using a large number of texture features. Moreover, texture features encompass various types, including GLCM, NGTDM,GLDM, etc. Multiple methods can be employed during feature extraction to obtain different types of texture features, which enhances prediction accuracy. By calculating all key features using eight algorithms, we found that the SVM algorithm yielded the highest diagnostic performance in the constructed predictive model (AUC=0.718). This may be due to the ability of the SVM algorithm to avoid overfitting issues to some extent, as it demonstrates robustness, stability, and good generalization ability, which are advantageous for high-dimensional data. The results of this study indicate that the radiomics model based on T2WI can accurately predict TB grading in rectal cancer, which is consistent with the findings of Li et al. (25). This could be attributed to the ability of radiomics to quantitatively analyze and link TB grading with the extracted image features, thus capturing the tumor heterogeneity factor that is indistinguishable to the naked eye through complex computational methods (35, 36).

No clinically significant risk factors related to TB grading (p>0.05) were found in this study, which is consistent with the conclusions of most studies (7, 34, 37). However, there is controversy surrounding these results, and one study has indicated that age is a clinical risk factor11. This discrepancy may be attributed to differences in the sampled population and sample size that could be collected.

The DCA indicates that the SVM model has the highest net clinical benefit, suggesting that the radiomics model developed may help in the formulation of personalized treatment management plans for patients. This finding is similar to the results of Li et al., who used a radiomics model based on multimodal MRI to predict tumor budding in locally advanced rectal cancer (25). Both studies utilized MRI-based radiomics models to predict tumor heterogeneity factors that cannot be identified by the naked eye. Furthermore, both studies demonstrated that the models can predict the TB grade. This may be attributed to the fact that radiomics models are based on medical imaging examinations, which provide digitized features of the tumor’s location. These models can identify tumor characteristics beyond the range of visual observation and imperceptible to the naked eye, and utilize machine learning algorithms to analyze, model, and predict these digitized features, thereby revealing the comprehensive situation of tumor growth and spread.

This study still has limitations. Firstly, as a retrospective study, it only included patients with rectal cancer who underwent curative surgery and had postoperative pathology indicating adenocarcinoma, resulting in selection bias. Secondly, there was sample bias, with a limited number of high-grade TB samples in the external validation group, leading to lower sensitivity. In future studies, it is necessary to increase the sample size. Thirdly, the area of interest was manually delineated, and subjective factors in the delineation process may have had some impact on the analysis, leading to subjective errors in the results. Fourthly, In terms of image segmentation, we devised our own exclusion criteria based on our experience regarding whether nodules closely connected to the tumor should be excluded as lymph node metastases. Although this exclusion criterion may not be completely rational, it improves the reproducibility in image segmentation. Fifthly,bowel preparation was not performed, which may have affected the quality and accuracy of the images.

In conclusion, our preliminary study suggests that the radiomics model based on T2WI can improve the preoperative prediction of tumor budding grading in rectal cancer and provide valuable reference for individualized treatment planning in clinical practice.

## Data availability statement

The original contributions presented in the study are included in the article/**Supplementary Material**. Further inquiries can be directed to the corresponding author.

## Ethics statement

The studies involving humans were approved by Qingdao Municipal Hospital Ethics Committee and Qingdao University Affiliated Hospital Ethics Committee. The studies were conducted in accordance with the local legislation and institutional requirements. The ethics committee/institutional review board waived the requirement of written informed consent for participation from the participants or the participants’ legal guardians/next of kin because This study is a retrospective study.

## Author contributions

XQ: Conceptualization, Data curation, Formal Analysis, Investigation, Methodology, Project administration, Resources, Validation, Visualization, Writing – original draft. LZ: Data curation, Formal Analysis, Methodology, Project administration, Resources, Writing – original draft, Writing – review & editing. WJ: Data curation, Funding acquisition, Writing – review & editing. JL: Conceptualization, Writing – review & editing. GW: Writing – review & editing, Conceptualization, Funding acquisition, Supervision, Writing – original draft.
