# Supplementary material for: Preoperative prediction of tumor budding in rectal cancer using multiple machine learning algorithms based on MRI T2WI radiomics
Source: Front Oncol. 2023 Oct 24;13:1267838. doi: 10.3389/fonc.2023.1267838 (PMC10628597; doi:10.3389/fonc.2023.1267838)
Supplement: Supplementary file 1 [file Table_1.docx]

Supplementary Material

# Supplementary Tables

Supplemental Table 1. The main sequences protocol and parameters of rectal MRI

| Hosptial | MRI machine | Magnetic field strength | TR | TE | Slice thickness | Interval | FOV | Sequence |
| --- | --- | --- | --- | --- | --- | --- | --- | --- |
| Hosptial 1 | Siemens Skyra | 3.0T | 6530 | 120 | 3 | 3.6 | 160×160 | TSE |
|  | GE Signa HDXT | 3.0T | 3240 | 107 | 4 | 4 | 180×180 | FSE |
| Hosptial 2 | GE Discovery 750 | 3.0T | 7270 | 110 | 3 | 3.6 | 200×200 | FSE |
|  | Siemens Skyra | 3.0T | 5000 | 100 | 3 | 3.6 | 180×180 | TSE |
|  | GE Signa HDXT | 3.0T | 4100 | 108 | 3.5 | 4 | 200×200 | FSE |
| Hosptial 3 | GE Discovery 750 | 3.0T | 10187 | 66 | 5 | 1 | 280×280 | FSE |

Hosptial 1 = Qingdao West Coast Hospital, Hosptial 2= Affiliated Hospital of Qingdao University,Hosptial 3= Qingdao Municipal Hospital.

Supplementary Table 2 Baseline characteristics of patients in cohorts

| Feature name | Train(Bd1&2) | Train(Bd3) | *P* value | Test(Bd1&2) | Test(Bd3) | *P* value |
| --- | --- | --- | --- | --- | --- | --- |
| age | 63.95±11.13 | 63.67±10.30 | 0.865 | 63.21±8.81 | 65.29±10.91 | 0.312 |
| DIS | 78.96±33.69 | 76.13±29.68 | 0.564 | 72.39±29.54 | 84.00±33.33 | 0.079 |
| length | 43.96±14.12 | 45.05±12.92 | 0.6 | 41.66±13.26 | 42.66±10.04 | 0.695 |
| deepth | 3.46±5.20 | 3.26±5.15 | 0.8 | 2.86±4.45 | 2.47±3.14 | 0.647 |
| sex |  |  | 0.024 |  |  | 0.777 |
| Female | 30(31.91) | 39(50.00) |  | 19(33.93) | 11(28.95) |  |
| Male | 64(68.09) | 39(50.00) |  | 37(66.07) | 27(71.05) |  |
| Cir |  |  | 0.292 |  |  | 1.0 |
| No | 46(48.94) | 31(39.74) |  | 24(42.86) | 16(42.11) |  |
| Yes | 48(51.06) | 47(60.26) |  | 32(57.14) | 22(57.89) |  |
| mrT |  |  | 0.519 |  |  | 0.498 |
| 1 | 6(6.38) | 2(2.56) |  | 2(3.57) | null |  |
| 2 | 17(18.09) | 13(16.67) |  | 17(30.36) | 9(23.68) |  |
| 3 | 65(69.15) | 60(76.92) |  | 30(53.57) | 25(65.79) |  |
| 4 | 6(6.38) | 3(3.85) |  | 7(12.50) | 4(10.53) |  |
| mrN |  |  | 0.517 |  |  | 0.088 |
| 0 | 51(54.26) | 47(60.26) |  | 24(42.86) | 13(34.21) |  |
| 1 | 25(26.60) | 21(26.92) |  | 19(33.93) | 21(55.26) |  |
| 2 | 18(19.15) | 10(12.82) |  | 13(23.21) | 4(10.53) |  |
| metastasis |  |  | 1.0 |  |  | 1.0 |
| No | 90(95.74) | 75(96.15) |  | 54(96.43) | 37(97.37) |  |
| Yse | 4(4.26) | 3(3.85) |  | 2(3.57) | 1(2.63) |  |
| CRM |  |  | 1.0 |  |  | 1.0 |
| No | 81(86.17) | 67(85.90) |  | 46(82.14) | 31(81.58) |  |
| Yse | 13(13.83) | 11(14.10) |  | 10(17.86) | 7(18.42) |  |
| EMVI |  |  | 0.535 |  |  | 1.0 |
| No | 75(79.79) | 66(84.62) |  | 46(82.14) | 32(84.21) |  |
| Yse | 19(20.21) | 12(15.38) |  | 10(17.86) | 6(15.79) |  |
| CEA |  |  | 0.404 |  |  | 0.507 |
| Normal | 60(63.83) | 44(56.41) |  | 36(64.29) | 21(55.26) |  |
| Abnormal | 34(36.17) | 34(43.59) |  | 20(35.71) | 17(44.74) |  |
| CA199 |  |  | 0.749 |  |  | 0.921 |
| Normal | 89(94.68) | 72(92.31) |  | 50(89.29) | 35(92.11) |  |
| Abnormal | 5(5.32) | 6(7.69) |  | 6(10.71) | 3(7.89) |  |

DIS: Distance from the tumor to the anal verge; Cir: Circumferential growth.;depth: depth of infiltration. *P*：Clinical characteristics of patients were compared using independent samples t-test, Mann-Whitney U test, or χ 2 test, *P* < 0.05 was considered statistically significant.
